# Supplementary figures and images for: Identifying and validating the presence of Guanine-Quadruplexes (G4) within the blood fluke parasite Schistosoma mansoni
Source: PLoS Negl Trop Dis. 2021 Feb 18;15(2):e0008770. doi: 10.1371/journal.pntd.0008770 (PMC7924807; doi:10.1371/journal.pntd.0008770)

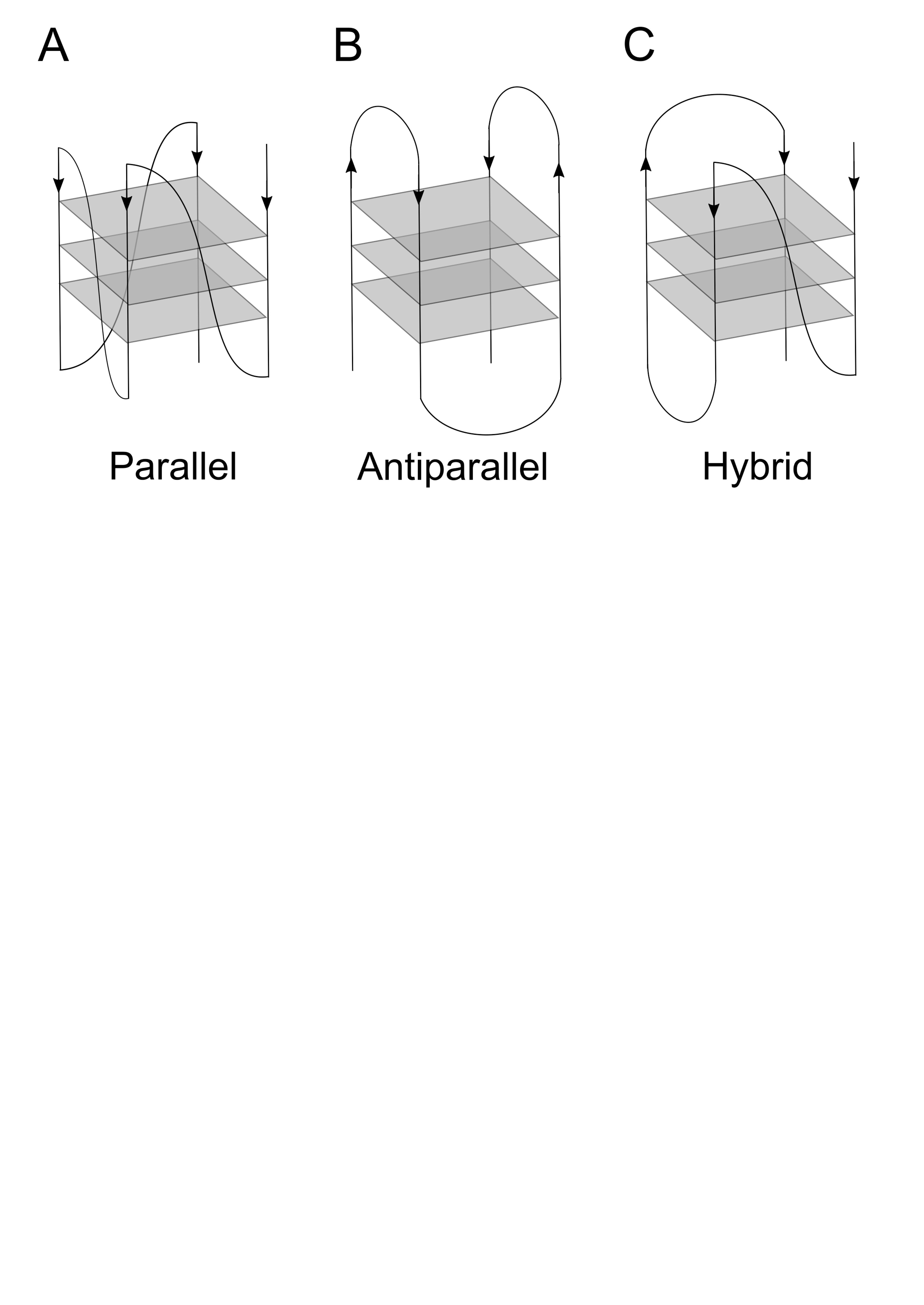

Supplement: S1 Fig — G4 have several topographical structures where strand directionality (arrowheads) and loop formation differ, affecting the tetrad each guanine forms (grey). A) In parallel G4 (e.g. H. sapiens c-myc), all four strands have the same directionality and loops form as “propellers” outwards from the stacked tetrads. B) In anti-parallel G4, strands have opposite directionality and loops form above as well as below the G4. C) In hybrid (3+1) G4, one strand runs anti-parallel to the rest and the loops are a mix of those found in parallel and anti-parallel G4 (e.g. H. sapiens hTelo). (TIF) [file pntd.0008770.s001.tif]

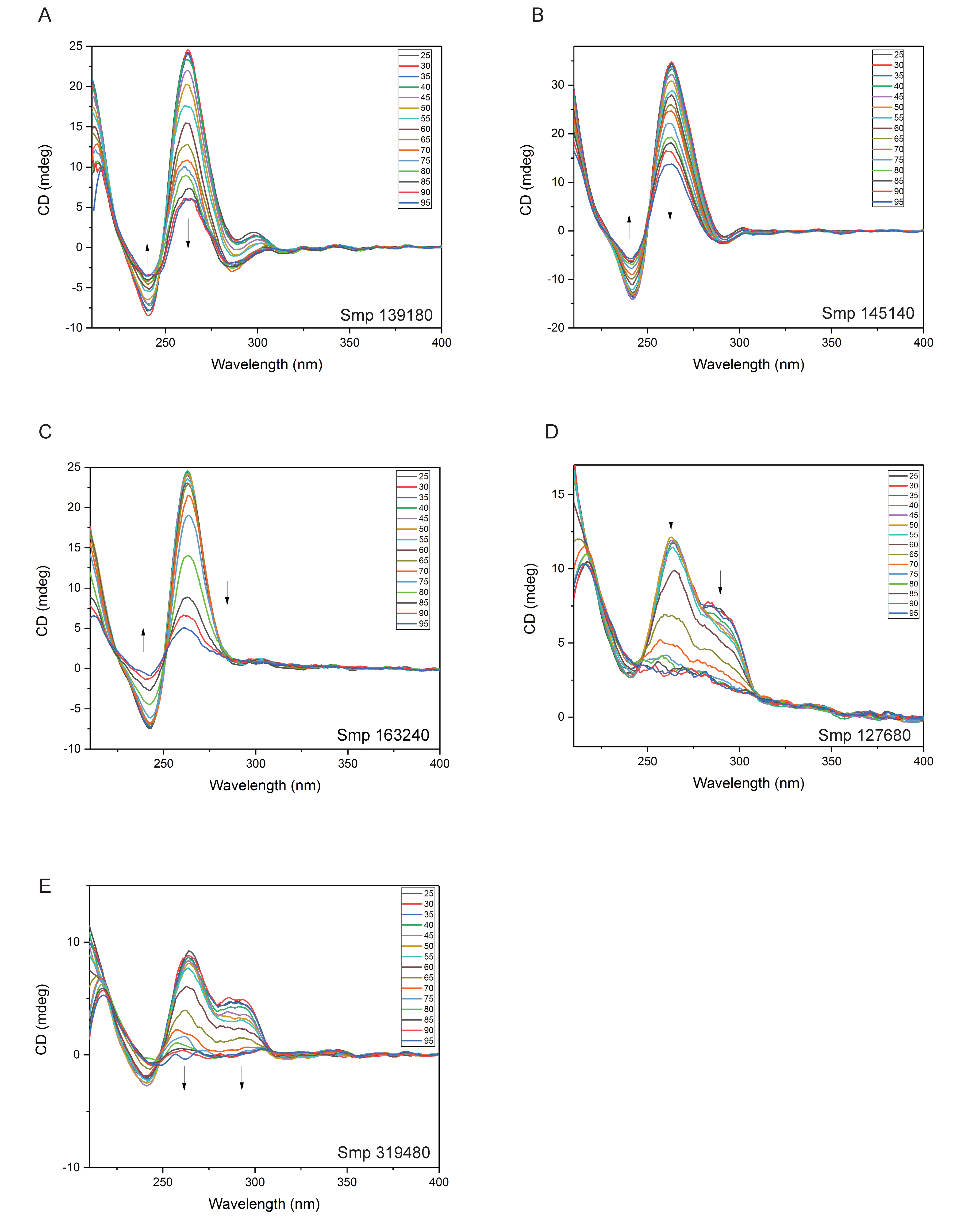

Supplement: S2 Fig — Oligos were adjusted to a 6 μM working solution in 60 mM TrisKCl (10 mM TrisHCl, 50 mM KCl; pH 7.4) buffer. CD spectra were recorded at 5°C intervals between 25°C and 95°C; data was used to calculate Tms. Parallel forming oligonucleotide sequences A) smp_139180, B) smp_145140 and C) smp_163240 showed stable structures that lost some ellipticity but not completely. Hybrid G4 folding oligonucleotide sequences D) smp_127689 followed the same trend as above whereas E) smp_319480 totally lost its ellipticity as temperature increased, indicating a complete unfolding of the G4. (TIF) [file pntd.0008770.s002.tif]

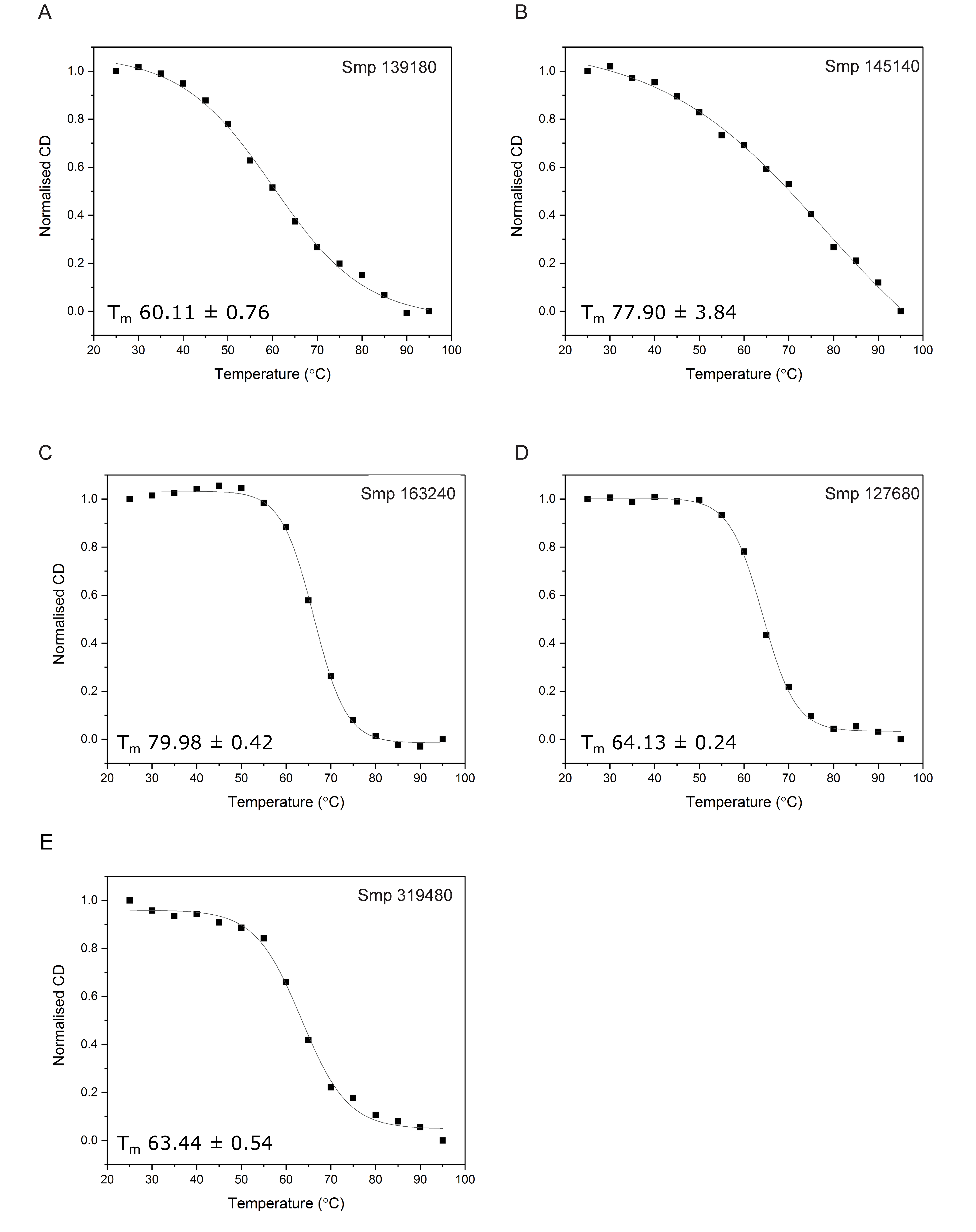

Supplement: S3 Fig — Spectra was recorded by CD at 5°C intervals between 95 and 25°C. Normalised CD was plotted, and Tm determined for each oligo sequence. Three repeats (average reading shown) were performed for each temperature point and regression analysis was performed to fit curves. (TIF) [file pntd.0008770.s003.tif]

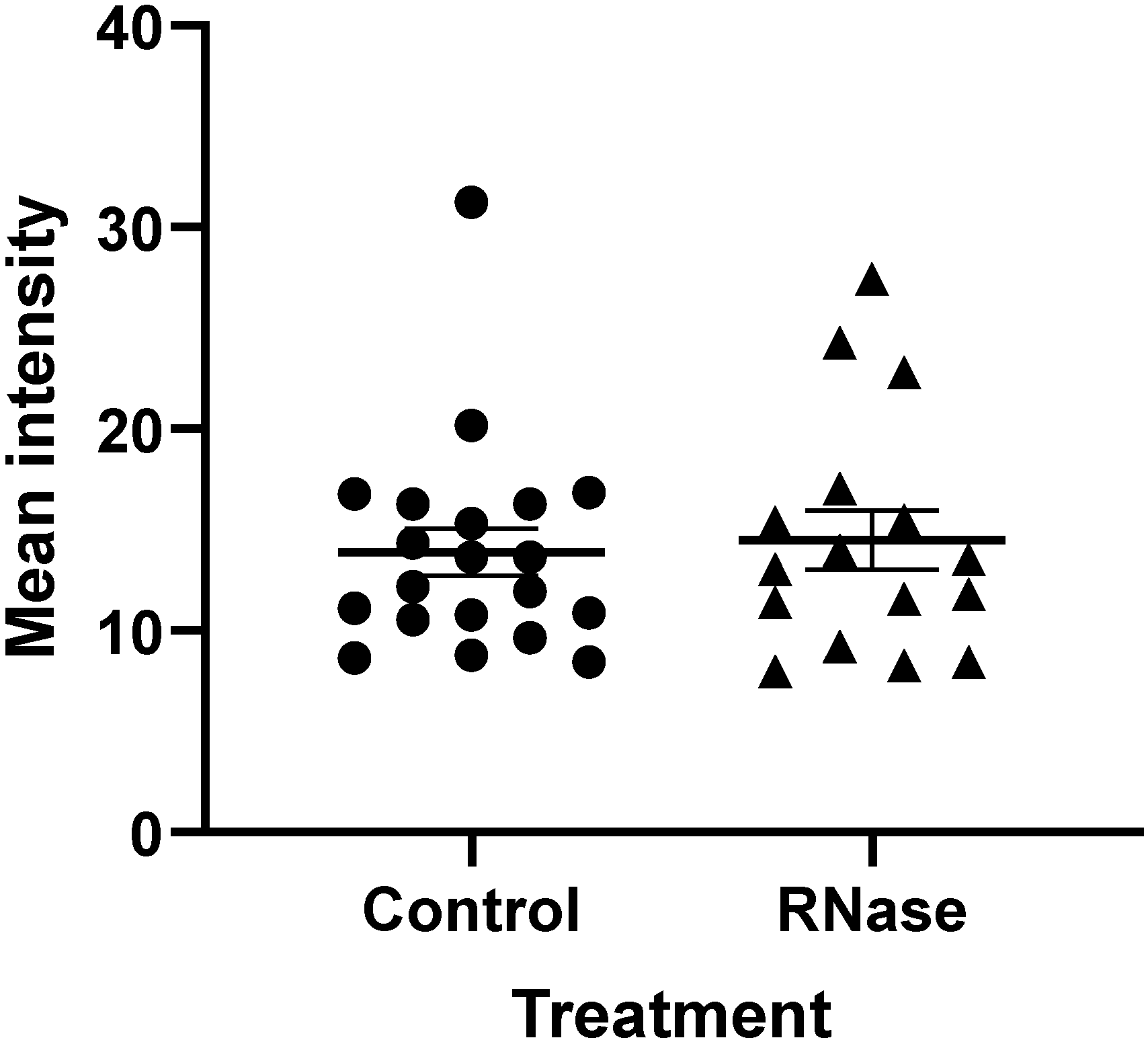

Supplement: S4 Fig — Mean signal intensities were acquired from LSCM images of male and female worms treated with enzymatically active RNase A (Fig 7) or buffer (enzyme free) controls (Fig 6). Mean intensity for each image were calculated by ImageJ and plotted as individual data points. Mean + SEM was plotted and Mann-Whitney t test was performed. No significance was observed between groups, indicating signal is not from RNA G4 structures. (TIF) [file pntd.0008770.s004.tif]

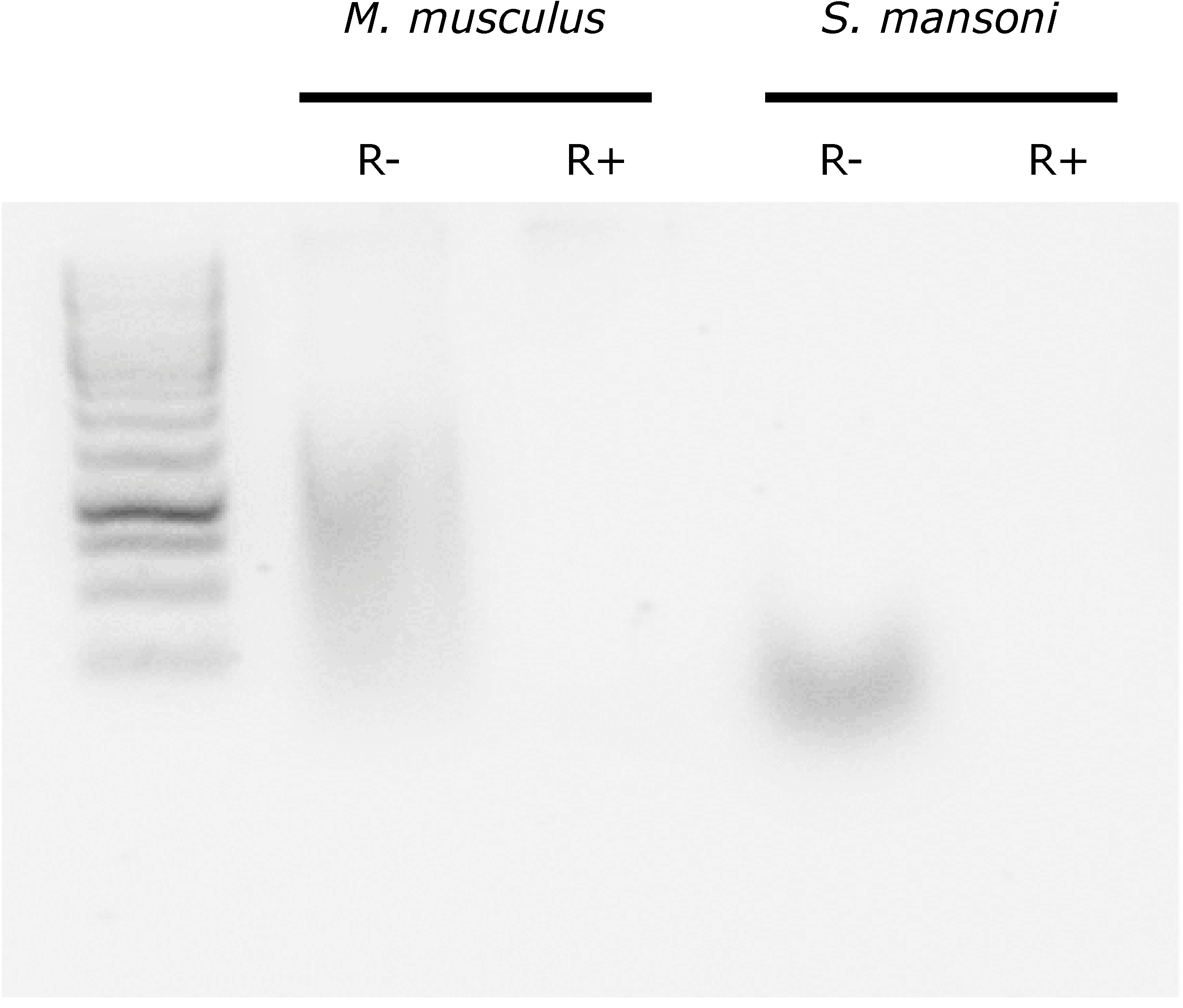

Supplement: S5 Fig — A sample of 1 μg each of total murine RNA and S. mansoni RNA were incubated for 1 hr in 0.1 mg/ml RNase (37 oC) and electrophoresed on an agarose gel with a 1kB ladder alongside RNA only controls (incubated under the same conditions in the absence of RNaseA). Complete degradation of host/parasite RNA was only observed in the presence of RNaseA (R+). (TIF) [file pntd.0008770.s005.tif]
