# Supplementary material for: Identifying and validating the presence of Guanine-Quadruplexes (G4) within the blood fluke parasite Schistosoma mansoni
Source: PLoS Negl Trop Dis. 2021 Feb 18;15(2):e0008770. doi: 10.1371/journal.pntd.0008770 (PMC7924807; doi:10.1371/journal.pntd.0008770)
Supplement: S2 Table — (DOCX) [file pntd.0008770.s007.docx]

**S2 Table. Number of telomeric and non-telomeric intergenic PQS per chromosome.**

| **Chromosome** | **Telomeric PQS** | **Non-telomeric Intergenic PQS** |
| --- | --- | --- |
| 1 | 19 | 10 |
| 2 | 30 | 9 |
| 3 | 22 | 17 |
| 4 | 9 | 8 |
| 5 | 81 | 7 |
| 6 | 18 | 13 |
| 7 | 3 | 9 |
| ZW | 7 | 22 |
